# Mental health in medical students during COVID-19 quarantine: a comprehensive analysis across year-classes

**DOI:** 10.6061/clinics/2021/e3007

**Published:** 2021-06-29

**Authors:** Thais Perissotto, Thamires Clair Rodrigues Pereira da Silva, Fabricio Petermann Choueiri Miskulin, Mariana Berwerth Pereira, Beatriz Astolfi Neves, Beatriz Cantieri Almeida, Amanda Victoria Casagrande, Salma Rose Imanari Ribeiz, Paula Villela Nunes

**Affiliations:** I Faculdade de Medicina de Jundiai, Jundiai, SP, BR.; II Departamento de Psiquiatria, Instituto de Psiquiatria (IPq), Hospital das Clinicas HCFMUSP, Faculdade de Medicina, Universidade de Sao Paulo, Sao Paulo, SP, BR.

**Keywords:** Medical Students, COVID-19, Anxiety, Depression, Empathy

## Abstract

**OBJECTIVES:**

The COVID-19 pandemic brought abrupt changes when quarantine measures were implemented. Most medical students had distance learning as their main content delivery mode, but in clerkship (fifth and sixth years), in-person activities were maintained under new protocols. These different modes may have affected student mental health. This study examines mental burden and empathy in medical students during the beginning of the COVID-19 pandemic according to the year of attendance.

**METHODS:**

All students attending first to the sixth year in the same medical school were invited to participate. The Hospital Anxiety and Depression Scale (HADS), the Self-Reporting Questionnaire (SRQ-20), the Interpersonal Reactivity Index (IRI), and the Mindful Attention Awareness Scale (MAAS) were provided.

**RESULTS:**

HADS scores for Anxiety and Depression (n=347) were 9.8±4.3 and 7.1±3.6, respectively; the SRQ-20 (n=373) score was 8.1±4.5; all scores were negatively correlated with the year of attendance. IRI (n=373) scores were: 2.6±0.5 (*Empathic Concern*), 2.7±0.7 (*Perspective Taking*), 2.5±0.9 (*Fantasy*), and 1.7±0.7 (*Personal Distress*). *Fantasy* was negatively correlated with the year of attendance. MAAS scores were positively correlated with the year of attendance. Worse mental health scores were found for first-year students across all scales.

**CONCLUSIONS:**

We found high levels of mental burden in medical students in the early period of the COVID-19 pandemic, especially in first-year students, who may have fewer resources to deal with stress. Moreover, as they entered college a short time before the pandemic, they were unable to experience academic life fully or create important new social support networks to deal with adversities.

## INTRODUCTION

The COVID-19 pandemic was a social stressor that could trigger or boost episodes of depression, anxiety, and other types of mental burden (1). Sources of stress were multiple, including the absence of definitive treatment for the disease, social isolation, and associated economic consequences. Different cohorts, including medical students, may have specific considerations during these challenging times (2). As for other healthcare workers, medical students, are in close contact with changes in health care systems when they occur, including those brought about by the COVID-19 pandemic.

Medical students face unique challenges during the pandemic, including disruption of pre-clinical and clinical training, adjustment to new social environments, particularly if social distancing requires a change of location, and exposure to high-risk environments (2). Many students had distance learning as their main course delivery mode, but some had maintained most of their in-person activities. In-person activities were especially retained in clerkship, but under new protocols and with increased safety measures.

Although the impact of the COVID-19 pandemic on healthcare workers has been well documented, the effects of this public health crisis on the mental health of medical students have not been sufficiently studied. The number of studies investigating the impact of the COVID-19 pandemic on mental health among medical students is rapidly increasing, but with inconsistent results. In some studies, stability was found (3-5), whereas increased anxiety and stress levels were observed in others, and depressive symptoms were less consistently altered (4,6). Predictors of worse mental health measures were higher levels of baseline depression or anxiety, presence of COVID-19 patients among family members or friends, and direct interactions with COVID-19 patients (6,7-9). Concern about the epidemic was associated with increased anxiety (8,9).

Differences were found in some studies when analyzing the mental burden across medical school years, and worse measures were observed among students attending earlier years (2,4,6,10-12). However, in some studies, clerkship students were not included, and few studies focused on first-year students (2,12) just entering medical school that had no time to establish bonds or a new social support network. Given this gap, the present study comprehensively analyzes mental health in medical students from the first to the sixth year during the first months of the 2020 COVID-19 pandemic in Brazil. We investigated Common Mental Disorders (CMD), depression, anxiety, empathy, and mindfulness levels.

## MATERIALS AND METHODS

In this cross-sectional study, all students from the first to the sixth year (680 students) of Jundiai Medical School were invited to participate. Student participation was voluntary, and students were not identified by name in the research. Those who signed the consent form were included in the study. The survey was conducted during the COVID-19 pandemic in Brazil, from March to June 2020, amidst strict quarantine measures. In this period, extracurricular activities were canceled, and students from the first to the fourth year (pre-clinical and clinical cycles) shifted to online learning. Students in the fifth and sixth year (clerkship) maintained their activities with enhanced safety measures such as smaller groups having contact with patients and protective clothing and equipment. In Brazil, a clerkship is an internship and is usually completed in a single institution, with no selection process.

Three shorter surveys were independently conducted for all students from the first to the sixth year to maximize participation. Each survey gathered information on gender, age, and year of attendance.

Survey #1 included the Hospital Anxiety and Depression Scale (HADS) (13). The HADS has two subscales: for anxiety (HADS-Anxiety) and depression (HADS-Depression), both ranging from 0 (absence) to 21 points. The recommended cut-off for screening clinically relevant symptoms is 9.

Survey #2 was designed to evaluate CMD and empathy. CMD was evaluated through the Brazilian validated version of the Self-Reporting Questionnaire (SRQ-20) (14). The World Health Organization developed the SRQ-20 to investigate nonpsychotic psychiatric disorders, screening for common mental disorders. The SRQ-20 comprises 20 items and includes questions regarding appetite, sleep, nervousness, unhappiness, tiredness, headaches, tremors, concentration, and other somatic complaints. The higher the score, the greater the likelihood of nonpsychotic mental disorders. The Interpersonal Reactivity Index (IRI) (15), Brazilian version (16), was applied to assess empathy. The IRI is considered one of the most reliable and valid self-assessed empathy measures along with the Jefferson Scale of Physician Empathy (17). The IRI includes 28 items to assess a multidimensional sense of empathy based on four subscales: *Empathic Concern*, *Perspective Taking*, *Fantasy,* and *Personal Distress*, each comprising seven items. *Personal Distress* evaluates self-focused responses to the suffering of others. *Empathic Concern* assesses situations that arouse feelings of compassion for others in distress. *Perspective Taking* assesses an individual’s ability to put themselves in the shoes of others, taking their perspectives. *Fantasy* assesses transposing oneself into fictional situations, exemplified as the tendency to play the role of fictional characters in books or films (15,16).

Survey #3 was designed to evaluate mindfulness levels. The Mindful Attention Awareness Scale (MAAS) (18), a Brazilian validated version, was used (19). The MAAS consists of 15 items answered on a Likert scale of 6 points, indicating how often participants experienced each of the situations described. The Likert scale ranges from 1 - almost always - to 6 - seldom. The final score is the result of adding up the answers and dividing the result by 15. Higher scores reflect higher levels of mindfulness. The final scores are categorized as “very poor” (scores 1.00-1.99), “poor” (2.00-2.99), “fair” (3.00-3.99), “good” (4.00-4.99), “very good” (5.00-5.99) and “excellent” (6.00). The MAAS is considered an effective scale for college (18) and medical students in Brazil (20).

Data from each survey were independently analyzed. Ordinal data were analyzed using the Chi-squared test. The Kolmogorov-Smirnov test was used to test for normal distribution. For gender comparisons and comparisons between years, the Kruskal-Wallis test was used, followed by the One-Way ANOVA using the Dunnett test for post-hoc testing. The first year is the reference category. Correlations were established using Spearman’s correlation test; the significance level was set at 0.05 in two-tailed tests. The Statistical Package for the Social Sciences (SPSS) version 20.0 was used to perform the statistical analyses.

This study was reviewed and approved by Jundiai Medical School Ethics Committee and is according to the Declaration of Helsinki.

## RESULTS

### Anxiety and depression (survey #1)

From the total sample, 347 students (51.0%) answered survey #1. In the survey #1 respondent group, the mean age was 22.6±2.7 years, and n=229 (65.9%) were women. HADS-Anxiety scores were 9.8±4.3, and the prevalence of HADS-Anxiety≥9 - above cut-off - was 59.7% (n=206). HADS-Depression scores were 7.1±3.6, and the prevalence of HADS-Depression ≥9 - above cut-off - was 36% (n=125). A difference in gender and age distribution across years was found (*p*<0.001 for both), as shown in Table 1.

There was an inverse correlation between year of attendance and HADS-Anxiety (rho=-0.215; *p*<0.001) and between the year of attendance and HADS-Depression (rho=-0.161; *p*=0.003).

**Figure 1:**
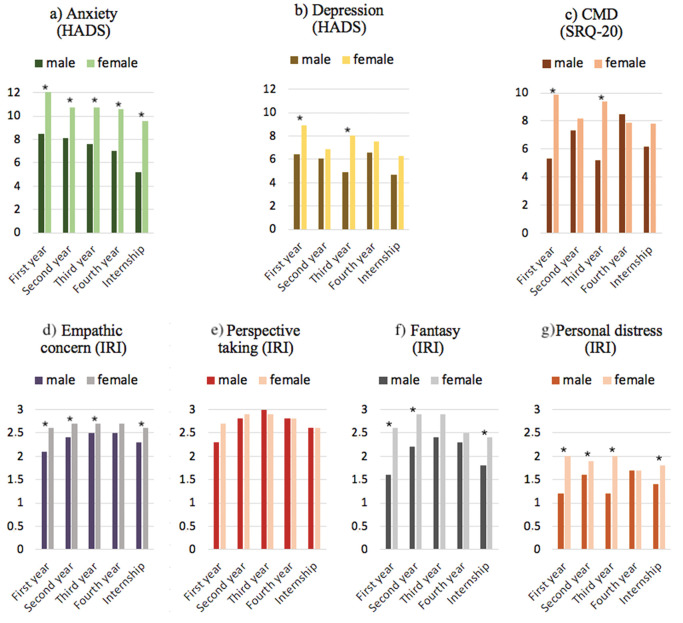
Comparison of mental health indices across the years and among genders. **Note**: SQR-20: self-reporting questionnaire with 20 items; HADS: The Hospital Anxiety and Depression Scale of Anxiety; IRI: Interpersonal Reactivity Index; Internship: clerkship, completed in the same institution. (*) *p*≤0.05 in Mann-Whitney test comparing male and female groups in each year year-class group.

Women had higher scores in the HADS-Anxiety than men, in the entire sample (11.0±3.9 and 7.5±4.1, respectively; *p*<0.001) and in each year of attendance: first year (*p*<0.001), second-year (*p*=0.029), third-year (*p*=0.016), fourth-year (*p*=0.001), and clerkship or internship (*p*<0.001), as shown in Figure 1. For HADS-Depression, women had higher scores than men in both the entire sample (7.9±3.5 and 6.1±3.6, respectively; *p*<0.001) and in the first year (*p*=0.003) and the third year (*p*=0.006), as shown in Figure 1.

When stratifying by gender, differences across years of attendance were found for women in HADS-Anxiety (*p*=0.031), and post-hoc tests revealed that first-year female students had higher scores than female clerkship students (12.2±4 and 9.6±3.4 respectively; *p*=0.006), as shown in Table 1 and Figure 1. Differences by year were also found in HADS-Depression (*p*=0.004) among women, with post-hoc tests finding that female first-year students had higher scores than female clerkship students (8.9±3.8 and 6.3±3.2, respectively; *p*=0.011), and higher scores than female second-year students (8.9±3.8 and 6.9±2.7, respectively, *p*=0.001). For men, no such differences were found.

### Common Mental Disorders and Empathy (survey #2):

From the total sample, 373 students (54.8%) answered survey #2. In the survey #2 respondent group, the mean age was 22.6±2.7 years, and n=271 (72.7%) were women. There was a difference in age distribution across years of attendance (*p*<0.001), and the difference in gender distribution was 0.064, as shown in Table 2.

Regarding CMDs, the SRQ-20 score was 8.1±4.5, and the prevalence of SRQ≥7 - above the cut-off for CMDs - was 60.1% (n=224). There was an inverse correlation between the year of attendance and SRQ-20 total scores (rho=-0.124; *p*=0.016).

Women had higher scores in the SRQ-20 than men across the entire sample (8.6±4.3 and 6.6±4.5, respectively; *p*<0.001), in the first year (*p*=0.005) and the third year (*p*=0.002), as shown in Figure 1. When stratifying by gender, a difference was found in SRQ-20 scores among years of attendance (*p*=0.049) for women, and post-hoc tests revealed that female first-year students had higher SRQ-20 scores than female clerkship students (9.9±4.3 and 7.8±4.2 respectively; *p*=0.027) as shown in Table 2 and Figure 1. For men, no differences were found for SRQ-20 scores.

In the IRI scale, we obtained the following scores: 2.6±0.5 (*Empathic Concern*), 2.7±0.7 (*Perspective Taking*), 2.5±0.9 (*Fantasy*), and 1.7±0.7 (*Personal Distress*). An inverse correlation between year of attendance and *Fantasy* was found (rho=-0.160; *p*=0.002). No correlation with the year of attendance was found for *Empathic Concern, Perspective Taking, or Personal Distress.* Women had higher scores than men in *Empathic Concern*, across the entire sample (2.7±0.5 and 2.4±0.5, respectively, *p*<0.001), and in the first year (*p*=0.005), second-year (*p*=0.026), third-year (*p*=0.040), and clerkship (*p*=0.001), as shown in Figure 1. For *Fantasy,* women had higher scores than men across the entire sample (2.7±0.9 and 2.1±0.9, respectively, *p*<0.001), and in the first year (*p*=0.002), second-year (*p*=0.005), and clerkship (*p*<0.001), as shown in Figure 1. For *Personal Distress*, women had higher scores than men across the entire sample (1.9±0.7 and 1.4±0.7, respectively, *p*<0.001), and in the first year (*p*=0.001), second-year (*p*=0.018), third-year (*p*=0.002), and clerkship (*p*=0.001), as shown in Figure 1. No gender differences were found for *Perspective Taking.* When stratifying by gender, we found differences in IRI subscales across years of attendance in *Fantasy,* both for men (*p*=0.040) and women (*p*=0.004). For men, post-hoc tests revealed that first-year students had lower scores than third-year students (1.6±0.7 and 2.4±1.1 respectively; *p*=0.047). No other differences were found for IRI scores.

### Mindfulness (survey #3):

From the total sample, 337 students (49.5%) answered survey #3, including the MAAS questionnaire. In the survey #3 respondent group, the mean age was 22.1±3.1 years, and n=213 (63.2%) were women. MAAS scores were on average 3.24±0.68 points, corresponding to a “fair” mindful level. The distribution of MAAS scores in our sample was: “very poor” n=10 (3.0%); “poor” n=100 (29.7%); “fair” n=179 (53.1%); “good” n=46 (13.6%); and “very good” n=2 (0.6%). MAAS scores were positively correlated with year of attendance (rho=0.149; *p*=0.006). There was a difference in age distribution across years of attendance (*p*<0.001) and no difference in gender distribution (*p*=0.212), as shown in Table 3.

Women had lower scores in the MAAS than men, considering the entire sample (3.5±0.7 and 3.1±0.7, respectively, *p*<0.001), and in the first year (*p*=0.003), as shown in Figure 1

When stratifying by gender, differences across years of attendance were found for women (*p*=0.008). Post-hoc tests revealed that female first-year students had lower MAAS scores than female third-year students (2.9±0.5 and 3.3±0.7 respectively; *p*=0.005) and lower MAAS scores than female clerkship students (2.9±0.5 and 3.3±0.7 respectively; *p*=0.026), as shown in Table 3 and Figure 1. For men, no differences were found.

## DISCUSSION

The present study is a comprehensive analysis of mental health among medical students from the first year to clerkship during the first months of the 2020 COVID-19 pandemic in Brazil. We investigated CMDs, depression, anxiety, and empathy and found high levels of mental burden, especially among first-year students. Most studies published so far have analyzed mental burden in medical students focused on depression or anxiety (2,4,6,21). Moreover, few studies have compared results of pre-clinical years with clinical years or clerkship. In our measures of CMDs, depression, and anxiety - SRQ-20 and HADS - a correlation with the year of attendance was found: the earlier the year, the greater the mental burden. For empathy, a similar correlation was found for the subscale *Fantasy*. Our study confirms high levels of mental burden in medical students during the COVID-19 pandemic (2,4,6-8,21-24), as has been observed in other population groups (8,22).

The fact that newcomers had the worst CMD scores may be related to several socio-environmental and behavioral factors. All students may have suffered from social isolation and online learning, but for first-year students, who shifted to remote learning two months after the beginning of classes, contact with the academic environment and the development of new social relationships in their Medical School setting were directly compromised by the pandemic. With such a short time to form bonds, establishing an emotional and social support network may have been compromised. For medical students, a social support network might positively affect mental health at times of adversity (25,26). Consistently, in China, a high prevalence of learning burnout was found among medical students during the COVID-19 epidemic period, and social support had a protective effect against stress (10). In Ireland, during the COVID-19 pandemic, medical students who felt supported by and who had confidence in their university had reported lower stress levels (11). It was reported that at the beginning of the pandemic, medical students in China relied more on social media than on scientific sources for obtaining information regarding COVID-19 than other health care workers (27). This information source could be more relevant among first-year students as they are less used to and less familiar with scientific sources. Health literacy (students’ ability to access, understand, appraise, and apply health information to healthcare, disease prevention, and health promotion) was found to protect medical students from fear during the COVID-19 pandemic (28). Health literacy has been recognized as a critical skill in evaluating online health-related information, especially in our increasingly digital world characterized by many sources of information (28). Therefore, taken together, lack of maturity, less resilience, weaker social support networks, and unreliable information may have been contributing factors for the inverse relationship found between the year of attendance and mental burden in our sample, as has been hypothesized in other studies (2,26,27). However, clerkship students had their routines differently affected by the COVID-19 pandemic and the strict quarantine measures adopted during the study period. In clerkship, most students could keep to their usual scheduled activities, despite some limitations imposed by safety measures. Additionally, they were not allowed to interact with COVID-19 patients directly, a known source of distress among health care workers (29) and medical students during the COVID-19 pandemic (12). Another interesting study conducted in Vietnam to validate a “fear of COVID-19” scale also found that senior students had less fear of COVID-19 (28).

Studies that investigated the impact of the COVID-19 pandemic on mental health among medical students are diverging. One study that analyzed mental health in university students according to the school year had results consistent with findings reported in this paper, despite not being conducted with medical students exclusively (9). Another study with 1,428 medical students, one of the largest samples seen, analyzed anxiety and depression in forty different American medical schools (2); 98% of that sample consisted of students from the first to the fourth year, and the study’s response rate was 9%. Consistent with our results, they found higher levels of anxiety and depression in pre-clinical years (first- and second-year students). Clerkship students, however, were not a significant percentage of their sample. In Turkey, in a sample of 3,105 medical students from 70 different medical schools, the prevalence of anxiety was the lowest among fifth-year students (equivalent to our clerkship students) (12). Two other smaller studies carried out exclusively with medical students did not find differences when comparing pre-clinical/clinical years with clerkship (4,6), but did not analyze first-year students specifically. In Japan, psychological distress was not different across medical school years during the COVID-19 pandemic, but it was associated with low self-esteem and self-efficacy (30). However, in a study with Chinese students, higher levels of burnout were found in senior students, but first-year students were not included (10). Nevertheless, in another study with university students during the COVID-19 outbreak in China that included medical students (41% of the sample) using the SRQ-20 for CMD measurement, age was a positive predictor of fewer distress symptoms (21).

Our study confirms high levels of mental burden in medical students during the COVID-19 pandemic (2,6-8,21-24). There are several possible reasons for these high levels, from changes in housing arrangements to fear of getting sick; uncertainty; and greater interest in media reports about the epidemic, among other factors (2,27). Known factors for worsened well-being among medical students are lack of time and conditions for study, sleep deprivation, lack of motivation to learn, excessive self-pressure for good grades, and lack of leisure time (25). Furthermore, for the medical students in the present study, most extracurricular activities were canceled with no remote alternative. In our sample, remote learning did not seem to be a major factor in explaining why first-year students had the worst mental health measures as all students from the first to the fourth years shifted to this modality of learning. In another similar prospective study, direct interaction with COVID-19 patients was one significant predictor of negative mental health measures (6). This finding might also explain why students in clerkship, who did not directly interact with COVID-19 patients, often had better levels of mental health than first-year students.

Consistent with research conducted before the pandemic, we also found higher CMDs, depression, and anxiety among women. There is plenty of evidence in the literature showing higher levels of depression and even higher levels of anxiety in women, both in overall populations (31), in health care workers (29), and medical students (32). Moreover, these results were confirmed in studies with medical students during the COVID-19 pandemic (4,6-9,12).

There are fewer studies on empathy, and none so far during the COVID-19 pandemic, even though empathy is increasingly recognized as a desired quality for medical students and health care workers (17,33). We found differences among school years for the IRI subscale *Fantasy*: the lower the year of attendance, the higher the scores (*p*=0.002), as was observed in our measures of CMD, anxiety, and depression. *Fantasy* might be related to the latter (17), but this correlation needs further examination. These results are not easy to read. *Fantasy* assesses transposing oneself into fictional situations (15,16), for instance, imagining oneself as a character or putting oneself in the shoes of patients or family members in the context of the COVID-19 pandemic. Thus, students from the first years had little clinical practice and might rely more on their imagination to interpret the current scenario. However, interns had much more clinical experience with critical care patients, and during the study period, had at least indirect contact with COVID-19 patients, despite new safety measures. This might have contributed to their lower scores on *Fantasy.*

As far as we know, this is the first study to evaluate mindfulness in medical students during the COVID-19 pandemic. Mindfulness is an ability that can be particularly helpful in disruptive and stressful situations such as the COVID-19 pandemic. Those that can achieve a mindful state are more “in tune” with their emotions and able to alter them better; such individuals are less likely to be self-conscious, socially anxious, or reflective (18). In our study, the measures of mindfulness were positively correlated to the year of attendance (rho=0.149; *p*=0.006). Few studies in the literature compare mindfulness levels among different age groups, especially among younger adults. Still, in a study that applied the MAAS in participants from middle-age to old age, older individuals had higher mindfulness scores than younger individuals (34). In our sample, we also found lower mindfulness levels for women (*p*<0.001), especially in the first year; higher levels of anxiety and depression were also found in this group. One possible explanation for lower mindfulness levels in younger female students may be that being mindful is negatively correlated with anxiety, automatic negative thoughts, and entanglement (35), often associated with anxiety and depression.

One of the main limitations of this study was the cross-sectional design of the research. Even though all students were individually invited to answer the questionnaires, there was also selection bias. The study was conducted with an online questionnaire. Our response rate was 50-55%, depending on the survey applied. Still, other published studies had response rates ranging from 9% to 31% (2,10,11). To be brief and thus improve participation rates, the scales used in this study were applied separately in surveys #1, #2, and #3; this, however, limits the comparison between scales, even though all scales were applied simultaneously. Another limitation in our study is the sample size of some subgroups. Studies in other medical schools, especially those that prospectively collect data, can help us evaluate whether these results were specific to stressful periods such as the COVID-19 pandemic and how enduring the related effects were.

## CONCLUSION

In this comprehensive analysis of mental health in medical students during the beginning of the COVID-19 pandemic, we found high levels of mental burden, especially in first-year students. First-year students entered medical school a short time before strict quarantine measures were imposed. Therefore, they were unable to experience academic life fully and create new social support networks to deal with adversities like the pandemic. In our CMDs, depression and anxiety (SRQ-20, HADS, respectively), a correlation with the year of attendance was found: the lower the year, the greater the mental burden. For empathy, a similar correlation was found for the subscale *Fantasy*. Our results also corroborate findings in the related literature regarding mental burden in medical students during the COVID-19 pandemic.

## AUTHOR CONTRIBUTIONS

Pereira MB, Casagrande AV and Nunes PV participated in conceptualizing the project and acquiring funding. Nunes PV designed the methodology and supervised the project. All of the authors participated in the investigation. Pereira MB, Almeida BC, Perissoto T and Nunes PV provided the data curation and formal analysis. Pereira MB and Nunes PV were responsible for the project administration. Pereira MB, Nunes PV, Almeida BC and Neves BA verified the underlying data. Pereira MB, Nunes PV, Almeida BC and Neves BA worked on visualization and wrote the original draft of the manuscript. Nunes PV, Pereira MB, Almeida BC Neves BA and Ribeiz SRI worked on writing the review and editing. All authors of the authors have participated sufficiently to take public responsibility for appropriate portions of the content

## Figures and Tables

**Table 1:** Analysis of HADS-Anxiety and HADS-Depression and with whom spend the quarantine in each year-class (n=347).

		First Year-class (n=102)	Second Year-class (n=63)	Third Year-class (n=66)	Fourth Year-class (n=69)	Internship (n=46)	*p*
Female		**71 (69.6%)^a^**	43 (68.3%)	51 (77.3%)	**30 (43.5%)^a^**	34 (73.9%)	**<0.001** ^b^
Age (years)		**20.2±1.9^a^**	20.7±1.3	**21.9±1.9^a^**	**23.7±3.7^a^**	**24.0±1.5^a^**	**<0.001** ^c^
HADS-Anxiety	Male	8.5±3.4	8.1±5.0	7.6±4.4	7.0±4.0	5.2±2.6	0.106^c^
	Female	**12.2±4.0**	10.7±4.2	10.7±3.9	10.6±3.7	**9.6±3.4^a^**	**0.031** ^c^
HADS-Depression	Male	6.4±3.5	6.1±4.3	4.9±3.7	6.6±3.5	4.7±2.8	0.362^c^
	Female	**8.9±3.8^a^**	**6.9±2.7^a^**	8.0±3.4	7.5±3.6	**6.3±3.2^a^**	**0.004** ^c^

**Note**: HADS Anxiety: The Hospital Anxiety and Depression Scale of Anxiety; HADS Depression: The Hospital Anxiety and Depression Scale of Depression; Internship: clerkship, completed in the same institution. (^a^) *p*≤0.05 in One-Way ANOVA post-hoc test using the first year as the reference category; (^b^) Qui-squared test - values are given as the number of cases and percentage of cases; (^c^) Kruskall-Wallis test - values are given as means±standard deviation. Bold values indicate significant differences.

**Table 2:** Analysis of self-reporting questionnaire with 20 items (SRQ-20) and Interpersonal Reactivity Index (IRI) of each year-class (n=373).

		First Year-class (n=65)	Second Year-class (n=58)	Third Year-class (n=75)	Fourth Year-class (n=54)	Internship (n=121)	*p*
Female		54 (83.1%)	39 (67.2%)	60 (80%)	36 (66.7%)	82 (67.8%)	0.064^a^
Age (years)		**20.5±2.3^b^**	20.9±1.5	**21.8±1.7^b^**	**23.4±2.6^b^**	**24.8±2.0^b^**	**<0.001^c^**
SRQ-20 total score	Male	5.3±4.4	7.3±5.0	5.2±4.5	8.5±4.3	6.2±4.2	0.181^c^
	Female	**9.9±4.3^b^**	8.2±4.2	9.4±4.1	7.9±4.8	**7.8±4.2^b^**	**0.027^c^**
SRQ items:							
Headaches	Male	0 (0%)	7 (36.8%)	3 (20%)	6 (33.3%)	12 (30.8%)	0.203^a^
	Female	22 (40.7%)	20 (51.3%)	27 (45%)	15 (41.7%)	35 (42.7%)	0.873^a^
Poor appetite	Male	3 (27.3%)	2 (10.5%)	3 (20%)	3 (16.7%)	2 (5.1%)	0.271^a^
	Female	18 (33.3%)	7 (17.9%)	17 (28.3%)	7 (19.4%)	13 (15.9%)	0.115^a^
Poor sleep	Male	4 (36.4%)	8 (42.1%)	3 (20%)	11 (61.1%)	19 (48.7%)	0.179^a^
	Female	**38 (70.4%)^b^**	23 (59%)	38 (63.3%)	**15 (41.7%)^b^**	42 (51.2%)	**0.049** ^a^
Easily frightened	Male	2 (18.2%)	7 (36.8%)	2 (13.3%)	3 (16.7%)	6 (15.4%)	0.341^a^
	Female	27 (50.0%)	15 (38.5%)	34 (56.7%)	11 (30.6%)	39 (47.6%)	0.112^a^
Hands shaking	Male	3 (27.3%)	6 (31.6%)	0 (0%)	3 (16.7%)	7 (17.9%)	0.189^a^
	Female	11 (20.4%)	10 (25.6%)	16 (26.7%)	5 (13.9%)	15 (18.3%)	0.535^a^
Nervousness	Male	7 (63.6%)	15 (78.9%)	10 (66.7%)	15 (83.3%)	31 (79.5%)	0.637^a^
	Female	**54 (100%)^b^**	39 (100%)	59 (98.3%)	**30 (83.3%)^b^**	**68 (82.9%)^b^**	**<0.001** ^a^
Poor digestion	Male	1 (9.1%)	4 (21.1%)	3 (20%)	7 (38.9%)	9 (23.1%)	0.443^a^
	Female	20 (37%)	15 (38.5%)	23 (38.3%)	10 (27.8%)	42 (51.2%)	0.148^a^
Difficult thinking	Male	3 (27.3%)	4 (21.1%)	4 (26.7%)	10 (55.6%)	13 (33.3%)	0.217^a^
	Female	25 (46.3%)	14 (35.9%)	32 (53.3%)	13 (36.1%)	33 (40.2%)	0.331^a^
Unhappiness	Male	4 (36.4%)	10 (52.6%)	3 (20%)	8 (44.4%)	20 (51.3%)	0.269^a^
	Female	38 (70.4%)	19 (48.7%)	37 (61.7%)	18 (50%)	44 (53.7%)	0.156^a^
Crying	Male	3 (27.3%)	5 (26.3%)	1 (6.7%)	3 (16.7%)	1 (2.6%)	**0.047** ^a^
	Female	20 (37%)	9 (23.1%)	21 (35%)	13 (36.1%)	25 (30.5%)	0.623^a^
Lack of enjoyment	Male	6 (54.5%)	10 (52.6%)	7 (46.7%)	13 (72.2%)	18 (46.2%)	0.455^a^
	Female	36 (66.7%)	24 (61.5%)	39 (65%)	17 (47.2%)	43 (52.4%)	0.214^a^
Difficulty making decisions	Male	3 (27.3%)	12 (63.2%)	11 (73.3%)	9 (50%)	17 (43.6%)	0.549^a^
	Female	37 (68.5%)	23 (59%)	36 (60%)	18 (50%)	54 (65.9%)	0.405^a^
Work suffering	Male	3 (27.3%)	4 (21.1%)	3 (20%)	8 (44.4%)	12 (30.8%)	0.509^a^
	Female	**20 (37%)^b^**	12 (30.8%)	23 (38.3%)	15 (41.7%)	**12 (14.6%)^b^**	**0.005** ^a^
Unable to be useful	Male	1 (9.1%)	3 (15.8%)	3 (20%)	8 (44.4%)	5 (12.8%)	0.058^a^
	Female	12 (22.2%)	8 (20.5%)	11 (18.3%)	4 (11.1%)	11 (13.4%)	0.542^a^
Loss of interest	Male	2 (18.2%)	7 (36.8%)	2 (13.3%)	8 (44.4%)	9 (23.1%)	0.214^a^
	Female	27 (50%)	11 (28.2%)	25 (41.7%)	13 (36.1%)	26 (31.7%)	0.154^a^
Worthlessness	Male	1 (9.1%)	7 (36.8%)	3 (20%)	7 (38.9%)	7 (17.9%)	0.195^a^
	Female	19 (35.2%)	13 (33.3%)	20 (33.3%)	10 (27.8%)	19 (23.2%)	0.537^a^
Suicidal thoughts	Male	**0 (0%)^b^**	**4 (21.1%)^b^**	0 (0%)	0 (0%)	1 (2.6%)	**0.010^a^**
	Female	6 (11.1%)	3 (7.7%)	2 (3.3%)	2 (5.6%)	3 (3.7%)	0.364^a^
Tiredness the all time	Male	4 (36.4%)	9 (47.4%)	7 (46.7%)	13 (72.2%)	20 (51.3%)	0.358^a^
	Female	**40 (74.1%)^b^**	23 (59%)	36 (60%)	27 (75%)	**37 (45.1%)^b^**	**0.004^a^**
Stomach ache	Male	2 (18.2%)	5 (26.3%)	3 (20%)	6 (33.3%)	12 (30.8%)	0.838^a^
	Female	28 (51.9%)	13 (33.3%)	25 (41.7%)	12 (33.3%)	37 (45.1%)	0.313^a^
Tires easily	Male	6 (54.5%)	11 (57.9%)	7 (46.7%)	12 (66.7%)	22 (56.4%)	0.848^a^
	Female	36 (66.7%)	19 (48.7%)	42 (70%)	28 (77.8%)	44 (53.7%)	**0.023^a^**
IRI:							
*Empathic Concern*	Male	2.1±0.5	2.4±0.5	2.5±0.4	2.5±0.5	2.3±0.5	0.448^c^
	Female	2.6±0.5	2.7±0.5	2.7±0.4	2.7±0.5	2.6±0.6	0.776^c^
*Perspective Taking*	Male	2.3±0.6	2.8±0.7	3.0±0.5	2.8±0.7	2.6±0.9	0.121^c^
	Female	2.7±0.6	2.9±0.6	2.9±0.6	2.8±0.6	2.6±0.6	0.131^c^
*Fantasy*	Male	**1.6±0.7^b^**	2.2±0.7	**2.4±1.1^b^**	2.3±0.9	1.8±0.7	**0.040^c^**
	Female	2.6±0.9	2.9±1.0	2.9±0.9	2.5±1.0	2.4±0.9	**0.004^c^**
*Personal Distress*	Male	1.2±0.9	1.6±0.7	1.2±0.6	1.7±0.7	1.4±0.6	0.156^c^
	Female	2.0±0.6	1.9±0.5	2.0±0.8	1.7±0.8	1.8±0.7	0.191^c^

**Note**: SRQ-20: self-reporting questionnaire with 20 items; IRI: Interpersonal Reactivity Index; Internship: clerkship, completed in the same institution. (^a^) Qui-squared test - values are given as the number of cases and percentage of cases; (^b^) *p*≤0.05 in One-Way ANOVA post-hoc test using the first year as the reference category; (^c^) Kruskall-Wallis test - values are given as means±standard deviation. Bold values indicate significant differences.

**Table 3:** Analysis of Mindful Attention Awareness Scale (MAAS) of each year-class (n=337).

		First Year-class (n=59)	Second Year-class (n=77)	Third Year-class (n=91)	Fourth Year-class (n=72)	Internship (n=38)	*p*
Female, n (%)		41 (69.5%)	46 (59.7%)	59 (64.8%)	39 (54.2%)	28 (73.7%)	0.212^a^
Age (years), mean±SD		**20.6±2.3^b^**	20.9±2.1	21.6±1.5	**23.9±4.5^b^**	**24.3±2.2^b^**	**<0.001** ^a^
MAAS total score	Male	3.6±0.7	3.4±0.8	3.4±0.5	3.4±0.6	3.7±0.7	0.542^c^
	Female	**2.9±0.5^b^**	3.0±0.7	**3.3±0.7^b^**	3.2±0.6	**3.3±0.7^b^**	**0.008^c^**

**Note**: MAAS: Mindful Attention Awareness Scale; Internship: clerkship, completed in the same institution. (^a^) Qui-squared test - values are given as the number of cases and percentage of cases; (^b^) *p*≤0.05 in One-Way ANOVA post-hoc test using the first year as the reference category; (^c^) Kruskall-Wallis test - values are given as means±standard deviation. Bold values indicate significant differences.
